# Effects of increasing the availability of vegetarian options on main meal choices, meal offer satisfaction and liking: a pre-post analysis in a French university cafeteria

**DOI:** 10.1186/s12966-024-01624-4

**Published:** 2024-07-16

**Authors:** Laura Arrazat, Claire Cambriels, Christine Le Noan, Sophie Nicklaus, Lucile Marty

**Affiliations:** 1 Centre des Sciences du Goût et de l’Alimentation, Institut Agro, CNRS, INRAE, Université de Bourgogne, Dijon, France; 2 Crous Bourgogne Franche-Comté, Dijon, France

**Keywords:** Choice architecture, Availability intervention, Food choices, Vegetarian meals, University cafeteria, Acceptability, Sustainability, University students

## Abstract

**Background:**

Changing the food environment is an important public health lever for encouraging sustainable food choices. Targeting the availability of vegetarian main meals served in cafeterias substantially affects food choice, but acceptability has never been assessed. We examined the effects of an availability intervention at a French university cafeteria on students’ main meal choices, meal offer satisfaction and liking.

**Methods:**

A four-week controlled trial was conducted in a university cafeteria in Dijon, France. During the two-week control period, vegetarian main meals constituted 24% of the offer. In the subsequent two-week intervention period, this proportion increased to 48%, while all the other menu items remained unchanged. Students were not informed of the change. Student choices were tracked using production data, and daily paper ballots were used to assess student satisfaction with the meal offer and liking of the main meal they chose (score range [1;5]). Nutritional quality, environmental impact, and cost of production of meal choices were calculated for each lunchtime. Food waste was measured over 4 lunchtimes during control and intervention periods. An online questionnaire collected student feedback at the end of the study.

**Results:**

Doubling availability of vegetarian main meals significantly increased the likelihood of choosing vegetarian options (OR = 2.57, 95% CI = [2.41; 2.74]). Responses of the paper ballots (*n* = 18,342) indicated slight improvements in meal offer satisfaction from 4.05 ± 0.92 to 4.07 ± 0.93 (*p* = 0.028) and in liking from 4.09 ± 0.90 to 4.13 ± 0.92 (*p* < 0.001) during control and intervention periods, respectively. The end-of-study questionnaire (*n* = 510) revealed that only 6% of students noticed a change the availability of vegetarian main meals. The intervention led to a decrease in the environmental impact of the main meals chosen, a slight decrease in nutritional quality, a slight increase in meal costs and no change in food waste.

**Conclusions:**

Doubling availability of vegetarian main meals in a university cafeteria resulted in a twofold increase in their selection, with students reporting being more satisfied and liking the main meals more during the intervention period. These results suggest that serving an equal proportion of vegetarian and nonvegetarian main meals could be considered in French university cafeterias to tackle environmental issues.

**Trial registration:**

Study protocol and analysis plan were pre-registered on the Open Science Framework (https://osf.io/pf3x7/).

**Supplementary Information:**

The online version contains supplementary material available at 10.1186/s12966-024-01624-4.

## Background

A shift toward sustainable diets is essential for meeting greenhouse gas emissions (GHGE) reduction goals and promoting healthier eating patterns [1–4]. Sustainable diets encompass four dimensions, as defined by the FAO and WHO [5, 6]: they should be nutritionally adequate, safe and healthy; respectful of the environment; economically fair; and culturally acceptable. In Western countries where the consumption of meat—a notoriously unsustainable food group—is high, the adoption of more plant-based diets is crucial to align with sustainable diet guidelines [1, 7–9].

There are numerous obstacles to reducing meat consumption, such as limited awareness of the impacts of meat on health and climate, insufficient cooking skills for plant-based meals, fear of stigma, high availability of meat products at relatively low prices, and low availability of vegetarian options in many out-of-home outlets [10, 11]. Overcoming such barriers requires diverse strategies to modify behavior [12, 13]. Two main types of interventions have been proposed [14, 15]: interventions targeting conscious determinants that raise individuals’ awareness (e.g., educational intervention targeting knowledge, skills, motivations), and structural interventions targeting the physical food environment which have been proven more efficient at promoting meat reduction than those targeting conscious determinants [12, 14, 16]. Educational interventions may increase the intention to reduce meat consumption [12, 14], but their effects on actual behavior may be countered by a physical environment promoting meat consumption through availability, placement, or pricing, resulting in an intention-behavior gap [17, 18]. Therefore interventions targeting meat consumption should primarily consider structural changes in the food environment [19, 20].

Among structural interventions, availability interventions increasing the variety or number of target food groups are a promising strategy for driving large-scale behavioral change [21–23]. For example, in an English university cafeteria doubling the availability of vegetarian meals from 25 to 50% led to a significant increase in the choice of vegetarian meals (41–79% depending on the experimental setting) [24]. However, these striking findings have never been replicated in the French context, where a strong preference for meat has been reported and vegetarian meal availability is still limited [25–27].

In French university cafeterias where the number of options served is limited and where students can only choose one main meal, the increased availability of one meal type comes at the expense of another, possibly replacing the preferred option [23, 28]. This raises concerns about the acceptability of availability interventions if the new food offer does not align with students’ preferences. Individuals are indeed more inclined to sustain a behavior over time if they derive pleasure from the new behavior [29]. However, none of the previous studies investigating the effects of increasing vegetarian meal availability have explored whether the change was acceptable for the participants [24, 30, 31]. Sekhon et al. proposed a theoretical framework for the acceptability of healthcare interventions comprising seven constructs: affective attitude, burden, perceived effectiveness, ethicality, intervention coherence, opportunity costs, and self-efficacy [32], highlighting that acceptability is a complex and multifactor concept. In the context of a food availability intervention, we suggest there are two key dimensions of acceptability: the level of satisfaction with the new food offer and the level of liking for the selected food.

We conducted a four-week controlled trial in a French university cafeteria where vegetarian main meal availability doubled during a two-week intervention period compared to a two-week control period. The first objective was to assess the impact of increasing the availability of vegetarian main meals on students’ main meal choices. We hypothesized that increased availability would lead to a greater selection of vegetarian main meal options. The second objective was to evaluate the acceptability of increasing the availability of vegetarian main meals by measuring students’ meal offer satisfaction and liking. We hypothesized a decrease in satisfaction and liking as vegetarian main meal availability increases because students’ preferred main meal options may have been removed. We triangulated the acceptability measure by quantifying food waste. As additional objectives, we investigated how the increased availability of vegetarian main meals affects students’ main meal choices in terms nutritional quality, carbon footprint and cost of production.

## Methods

### Study design

**Fig. 1:**
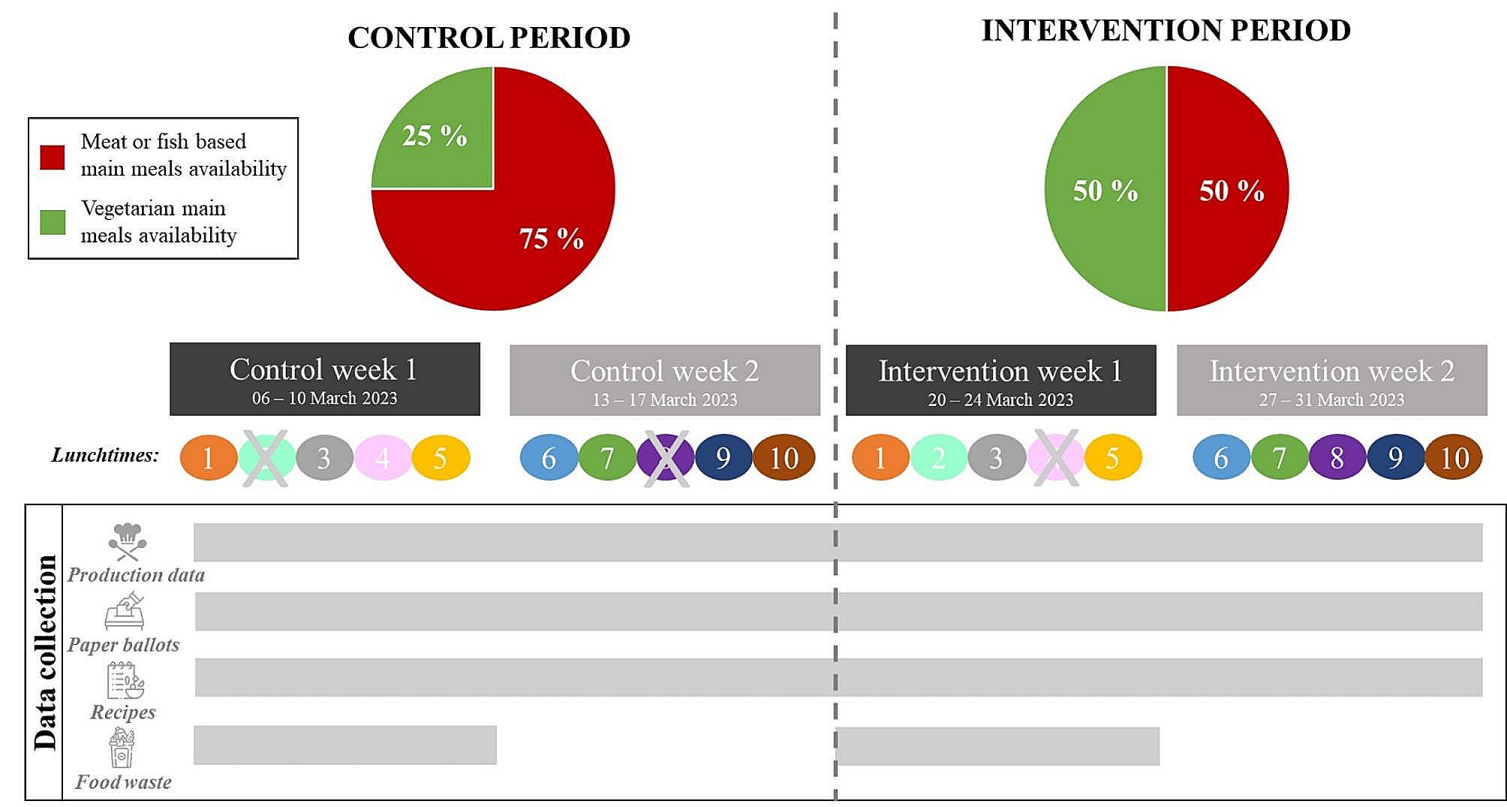
Experimental design for modification of the availability of vegetarian meals intervention and data collection procedure Legend: Grey crosses depict days when data collection could not be carried due to strike actions led by the university cafeteria staff. The same food items were offered on days with the same color and number in the control and intervention period (except for the increase in vegetarian main meals) Production data: Vegetarian main meal availability (%) and percentage of chosen vegetarian main meals (%) Paper ballots: Satisfaction score (range [1;5]) and liking score (range [1;5]) Recipes: Nutritional quality, environmental impact and cost of production of chosen main meals Food waste: Weight of leftovers on the plates

A four-week controlled experiment was conducted during lunch hours in a university cafeteria (Dijon, France), serving approximately 2000 meals per lunchtime. The experiment comprised two phases (Fig. 1): a two-week control period with the aim of serving 25% of vegetarian main meals consistently with the observed availability in this cafeteria before the experiment and a two-week intervention period with the aim of serving 50% of vegetarian main meals. The vegetarian main meals excluded meat and fish but might include eggs and/or dairy products. The data were collected during both the control and the intervention periods to conduct pre-and post-data analysis.

Both the control and intervention periods included ten lunchtimes matched for recipes of the main meals, starters, and desserts to obtain ten “control-intervention” paired lunchtimes. Doubling the proportion of vegetarian main meals was achieved both by increasing the number of servings for each vegetarian main meal and by substituting one meat based main meal with a vegetarian main meal. Between 1 and 2 vegetarian options were served per lunchtime during the control period, and between 2 and 3 during the intervention period but the initial number of options remained similar (*n* = 5 or 6) resulting in a net reduction of meat options. All the menus for the main meals served during the experiment are presented in supplementary materials (S1). During the four weeks of the experiment, we ensured that a vegetarian and a nonvegetarian main meal option were always available until the end of the lunchtime. Students were not informed of the change in the food offered before or during the experiment and were not compensated for their participation. The study design was initially proposed by the research team and then discussed and codeveloped with the university cafeteria staff; details on the co-construction process are provided in supplementary materials (S2). The protocol resulting from this co-construction process was approved by the CEEI-IRB ethical committee for research (reference: n◦23–976, delivered on January 10th, 2023).

### Measures

#### Main meal choices

The university cafeteria was on 3 floors and 5 or 6 different main meals were served every day, all at the same price for the students. The students could only choose one of them. At the entrance, a digital board displayed the description of all the 6 main meals and at which floors they were available. The university cafeteria staff were free to serve the vegetarian main meals on the different floors which may vary from one day to another. On each floor, the students could see the available options in trays and were served to order.

Every lunchtime during the experiment and for each main meal option, university cafeteria staff recorded the number of servings produced and servings remaining. The availability of vegetarian main meals was calculated as the number of vegetarian servings produced over the total number of servings produced (in %) which represents the average probability of vegetarian meals availability across one lunchtime. The number of vegetarian and nonvegetarian main meal choices was calculated as the difference between the number of servings produced and servings remaining for each main meal option.

#### Main meal offer satisfaction and liking

Every lunchtime during the experiment, anonymous paper ballots were handed out to all the students who ate at the university cafeteria by a team of five experimenters at the checkout counters. Students were asked to answer the questions on the ballots and to place them back in a box near the cafeteria exit. Alternatively, students had the option to answer the same questions online using QR codes positioned on each cafeteria table and linked to the Lime Survey© platform. Students were informed orally and through posters that we were conducting a study regarding how much they liked the food at the university cafeteria and were free to decide whether to take part or not for each lunchtime. The ballot design is presented in the supplementary materials (S3). The survey comprised four questions: “Are you satisfied with the food that was offered today?”, ranging from 1 “not at all satisfied” to 5 “very satisfied” (meal offer satisfaction score) and “Which main meal did you have today?” to select from the list of the main meals available on the day, “How much did you like the main meal you had today?” on a scale ranging from 1 “I did not like it at all” to 5 “I truly liked it” (liking score) and “At what time were you served?”.

#### Food waste

Food waste was measured on a subsample of lunchtimes (*n* = 8, 4 during the control period and 4 during the intervention period) as an additional, nondeclarative measure of the acceptability of the increased availability of vegetarian main meals. Previous research has indicated that food waste is a reliable indicator of meal preferences and overall satisfaction with food [33, 34]. During the first and third experimental weeks, university cafeteria staff members asked all the students to place leftovers from their main meals, starters/desserts, and breads into three separate bins. Food weight data (in g/day) were recorded by university cafeteria staff.

#### Sustainability indicators

Measures of satisfaction, liking and food waste address the sociocultural aspect of sustainability, but food sustainability also includes nutritional, environmental, and socioeconomic dimensions [5, 6]. To assess whether increasing the availability of vegetarian main meals improved the overall sustainability of students’ food choices and fill a research gap regarding those aspects [31], we calculated three indicators: nutritional quality, GHGE and cost of production. They were calculated based on actual recipes for each main meal retrieved from the university cafeteria informatic system. For each lunchtime, we computed indicators of nutritional quality, environmental impact and cost averaged across all main meals weighted by the number of meals chosen. All the indicators for each main meal are given in supplementary materials (S4).

##### Nutritional quality

To estimate the nutritional composition of each of the ingredients composing the 63 main meals served during the experiment, we used two French food nutrient reference tables: Ciqual 2020 and Calnut 2020. We then assessed the nutritional quality of the main meals using the FSA score (the British Food Standards Agency nutrient profiling system) based on the allocation of positive and negative points for respectively unfavorable nutrients (energy, saturated fatty acids, total sugar and sodium) and favorable nutrients or food groups (protein, fiber, fruits, vegetables, legumes and nuts), respectively, and calculated the FSA score per 100 g [35]. We used the updated version of the FSA algorithm and obtained scores ranging from − 17 to 55, with higher scores indicating poorer nutritional quality [36].

To determine the average nutritional quality of the choices made during one lunchtime, we calculated the average FSA score for all main meals served during this lunchtime weighted by the number of servings actually chosen (i.e., production - leftovers).

##### Environmental impact

The environmental impact of the main meals was estimated as the GHGE because they are strongly correlated with the Environmental Footprint single score, an aggregated environmental impact score developed by the European Commission [37]. Additionally, GHGE are the most reliable environmental indicator available in French food databases [38]. The GHGE data for each of the ingredients composing the main meals served during the experiment were retrieved from the Agribalyse database [38], the French food environmental impact database based on life cycle analysis drawn up by the French Agency for Ecological Transition, which includes GHGE values in kg of CO_2_e/kg for 2480 foods. We then calculated the GHGE for one serving of each of the 63 main meals.

To determine the average environmental footprint of the choices made during one lunchtime, we calculated the average GHGE for all main meals served during this lunchtime weighted by the number of servings actually chosen (i.e., production - leftovers).

##### Cost of production

The cost (in €) of one serving of a main meal was computed automatically on the university cafeteria’s informatic system and was retrieved for the 63 main meals served. From these data, we determined the average cost of the choices made during one lunchtime by calculating the average cost for all main meals served during this lunchtime weighted by the number of servings actually chosen (i.e., production - leftovers).

#### Additional measures: feedback questionnaire

During the last two lunchtimes of the study (March 30th and 31st, 2023) and the following lunchtime (April 3rd, 2023), we handed out QR codes linked to an anonymous online questionnaire to the students in the university cafeteria. The eligibility criteria were to 1/have eaten at the university cafeteria during the experimental period, 2/be older than 18 years of age and, 3/be a student. The questionnaire was hosted on the Lime Survey© platform and lasted approximately 5 min. Students were asked for their feedback on the food offered during the past two weeks. We assessed whether they had noticed any changes by asking them “Have you noticed any changes in the main meals offered during lunchtimes over the past two weeks?“. If the participants responded affirmatively, then we asked them to describe what they had observed using free text. Two independent researchers coded whether a participant guessed that there was a change regarding vegetarian main meals. We then told the participants that there was a twofold increase in the availability of vegetarian main meals during the last two weeks and asked them to indicate whether they had the impression of having eaten more vegetarian main meals; if so, we asked them to rate the level of constraint on a scale from 1 (“not burdensome at all”) to 10 (“highly burdensome”). Finally, sociodemographic characteristics were measured: age, sex, scholarship status, type of educational institution, field of study, highest educational qualification obtained, place of living and declared diet (i.e., omnivorous, flexitarian, pesco-vegetarian, ovo-lacto-vegetarian, or vegan).

### Statistical analyses

We followed a preregistered analysis plan (https://osf.io/pf3x7/) and slight deviations are explained below. All analyses were conducted for 17 lunchtimes, as data collection could not be carried out for two lunchtimes during the control period and one lunchtime during the intervention period due to strike actions. We first analysed the effect of the intervention (predictor: control, intervention) on the choice of vegetarian main meals by students (outcome: 0 for non-vegetarian, 1 for vegetarian, binomial distribution) using a generalized linear mixed model that included lunchtimes as a random effect to consider the pairing between the control and intervention periods (*n* = 10). For descriptive purposes, we calculated and represented the correlation between the availability of vegetarian main meals (%) and vegetarian main meal choices (%) for each lunchtime (*n* = 17). Due to strike actions that led to only having seven pairs of lunchtimes, we will not report results from the preregistered secondary analysis, which aimed to estimate the relationship between the increase in the availability of vegetarian main meals and the increase in the odds of choosing a vegetarian main meal.

We then evaluated the effect of the intervention (predictor: control, intervention period) on students’ meal offer satisfaction and liking (outcomes: scores from 1 to 5) using generalized linear mixed models that included lunchtimes as a random effect. Finally, to test whether the intervention differentially affected the liking of vegetarian and nonvegetarian main meals, we analyzed the effect of the intervention (predictor: control, intervention period), the type of main meal (predictor: vegetarian, nonvegetarian) and the interaction between the intervention and the type of main meal on students’ liking (outcome: score from 1 to 5) using a generalized linear mixed model. A random effect of the main meal recipe (e.g., cheese omelet, pork curry) was added to the model to consider the clustering of liking data for the same recipe across lunchtimes. As satisfaction and liking data were obtained from a subsample of students, we verified whether the main meal choices reported on the ballots were representative of the main meals served each lunchtime with chi-square tests. Regarding food waste data (exploratory analyses, not preregistered), we compared average food waste from the main meal between the control and intervention periods using a nonparametric Wilcoxon comparison test (*n* = 8). Finally, as secondary analyses, we compared the nutritional quality, environmental impact and cost of the main meals chosen between the control and intervention periods using generalized linear mixed models, with intervention as a predictor (control, intervention period) and lunchtime as a random effect.

All the statistical analyses were performed using SAS version 9.4 (SAS Institute, Inc., 2012 SAS^®^ 9.4. Cary, NC) and the level of significance was set at *p* < 0.05 for all of the analyses.

### Sample size rationale

We chose to carry out a four-week study for logistical reasons because we wanted to ensure uninterrupted data collection in the university cafeteria with minimal fluctuations in student attendance. This meant excluding exam periods and school holidays from our study. Additionally, we limited the intervention period to two weeks, as during the co-construction process (described in supplementary materials (S2)), this was mentioned as the longest acceptable timeframe for the chefs to conduct this type of intervention. On each study lunchtime, we expected an attendance of ~ 2,000 students, given the usual sales data for this cafeteria.

## Results

### Participants

**Fig. 2:**
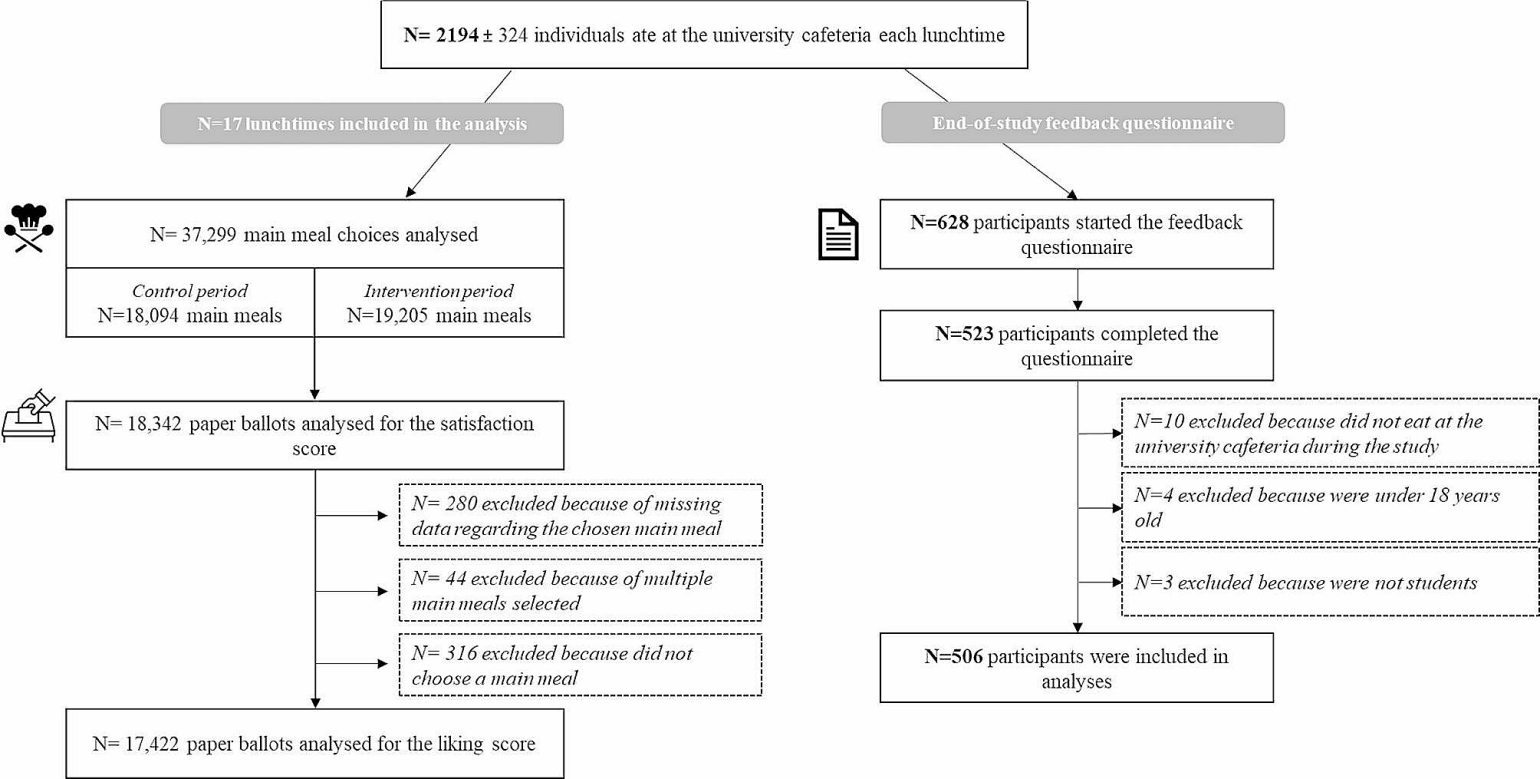
Study flow chart for production and paper/online ballot survey data and end-of-study feedback questionnaire

During this 4-week experiment, we collected data on 37,299 main meal choices over 17 lunchtimes for an average of 2194 ± 324 (mean ± standard deviation) students per lunchtime, with 18,094 main meal choices during the 2-week control period and 19,205 during the 2-week intervention period. Across the 17 lunchtimes, 54% of the students participated in the daily satisfaction and liking survey for a total of 18,342 responses to be analyzed, as shown in the study flow chart (Fig. 2). These respondents accurately represented overall students’ main meal choices, as we found no difference in the percentage of vegetarian main meal choices overall (university cafeteria data) or in this subsample (ballot data) during either the control or the intervention period (Table 1).

**Table 1:** Representativeness of the ballot survey answers

	Production data. Percentage of vegetarian main meals choosen	Ballot survey. Percentage of ballots with a vegetarian main meal	Chi-2 (*p*-value)
Control period	23 ± 7%	22 ± 8%	0.05 (*p* = 0.824)
Intervention period	45 ± 16%	45 ± 15%	0.30 (*p* = 0.584)

Values are means ± standard deviations

### Effect of the intervention on students’ food choices

**Fig. 3:**
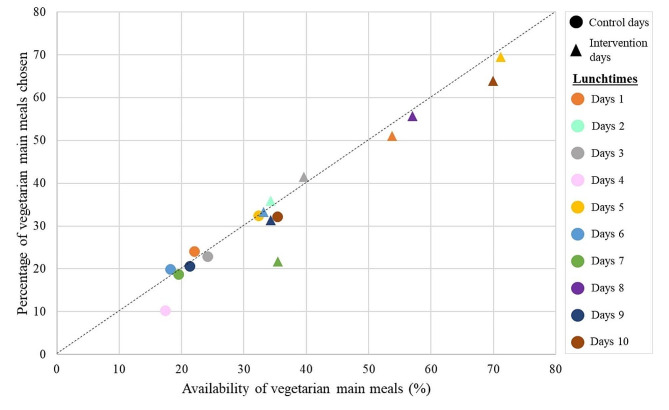
Scatter plot representing the correlation between availability and percentage of choice of vegetarian main meals

For the 17 lunchtimes included in the study, we observed a significant and strong correlation between the availability of vegetarian main meals and the choice of vegetarian main meals (*r* = 0.97, *p*-value < 0.001) (Fig. 3). The availability of main vegetarian meals increased from 24% during the control period to 48% during the intervention period, leading to a significant increase in the likelihood of vegetarian main meal choices: OR = 2.57, 95% CI = [2.41;2.74]. Indeed, 23% of the participants consumed vegetarian main meals during the control period representing sales of 502 ± 135 vegetarian meals and 1760 ± 325 non-vegetarian meals per day, and 45% of the participants consumed vegetarian main meals during the intervention period representing sales of 945 ± 316 vegetarian meals and 1189 ± 397 non-vegetarian meals per day.

### Effect of the intervention on students’ meal offer satisfaction and liking and on food waste

**Fig. 4:**
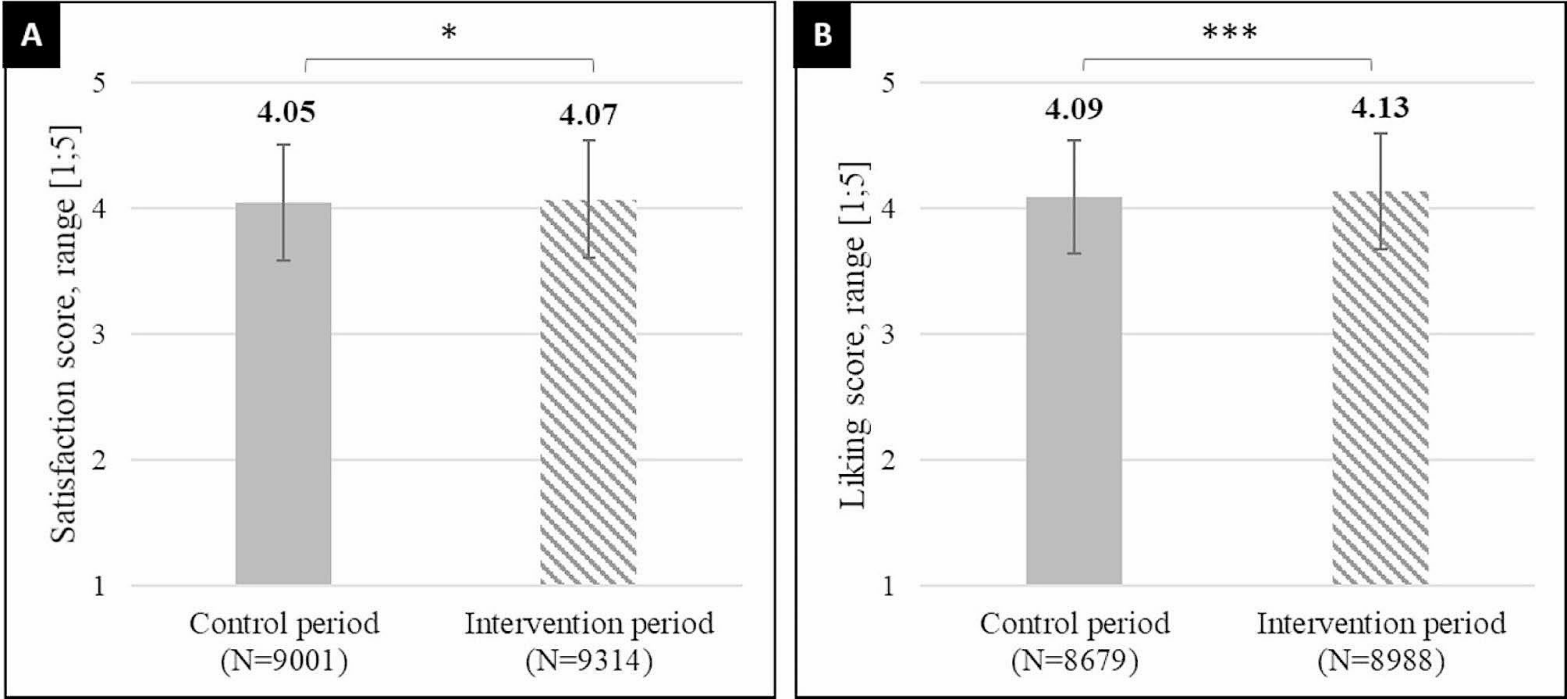
Satisfaction (**A**) and liking (**B**) scores during the control and intervention periods Legend: Error bars are standard deviations

A twofold increase in the availability of vegetarian main meals at the university cafeteria led to a significant, yet slight, increase in satisfaction with the food offered from 4.05 ± 0.92 to 4.07 ± 0.93 (β = 0.03, t(18,341) = 2.20, *p* = 0.028), as shown in Fig. 4A. Similarly, we observed a significant but slight increase in the liking score of the main meals chosen by the students, from 4.09 ± 0.90 to 4.13 ± 0.92 (β = 0.07, t(17,421) = 4.57, *p* < 0.001), as shown in Fig. 4B. Satisfaction and liking scores for each day are reported in supplementary materials S5 and S6, respectively, and average liking scores for each of the 63 main meals served during the study are reported in supplementary materials S4.

A linear mixed model allowed us to determine that the average liking score for vegetarian main meals (4.01 ± 1.01) was slightly lower than that for nonvegetarian main meals (4.17 ± 0.85) (β=-0.14, t(17,421)=- 2.05, *p* = 0.009). However, we did not observe an effect of the interaction between the type of main meal (vegetarian or nonvegetarian) and the intervention of the study on the liking score (β=-0.05, t(17,421)=-1.38, *p* = 0.168), meaning that the vegetarian options chosen during the control and the intervention periods were similarly liked by the students.

Finally, the results from food waste observations (*n* = 8 lunchtimes) revealed that the average quantity of food waste per student (meals, starters and desserts) was 36.0 ± 12.9 g during the control period and 34.4 ± 4.9 g during the intervention period (*p* = 0.50).

### Effect of the intervention on the sustainability of the main meals chosen

**Fig. 5:**
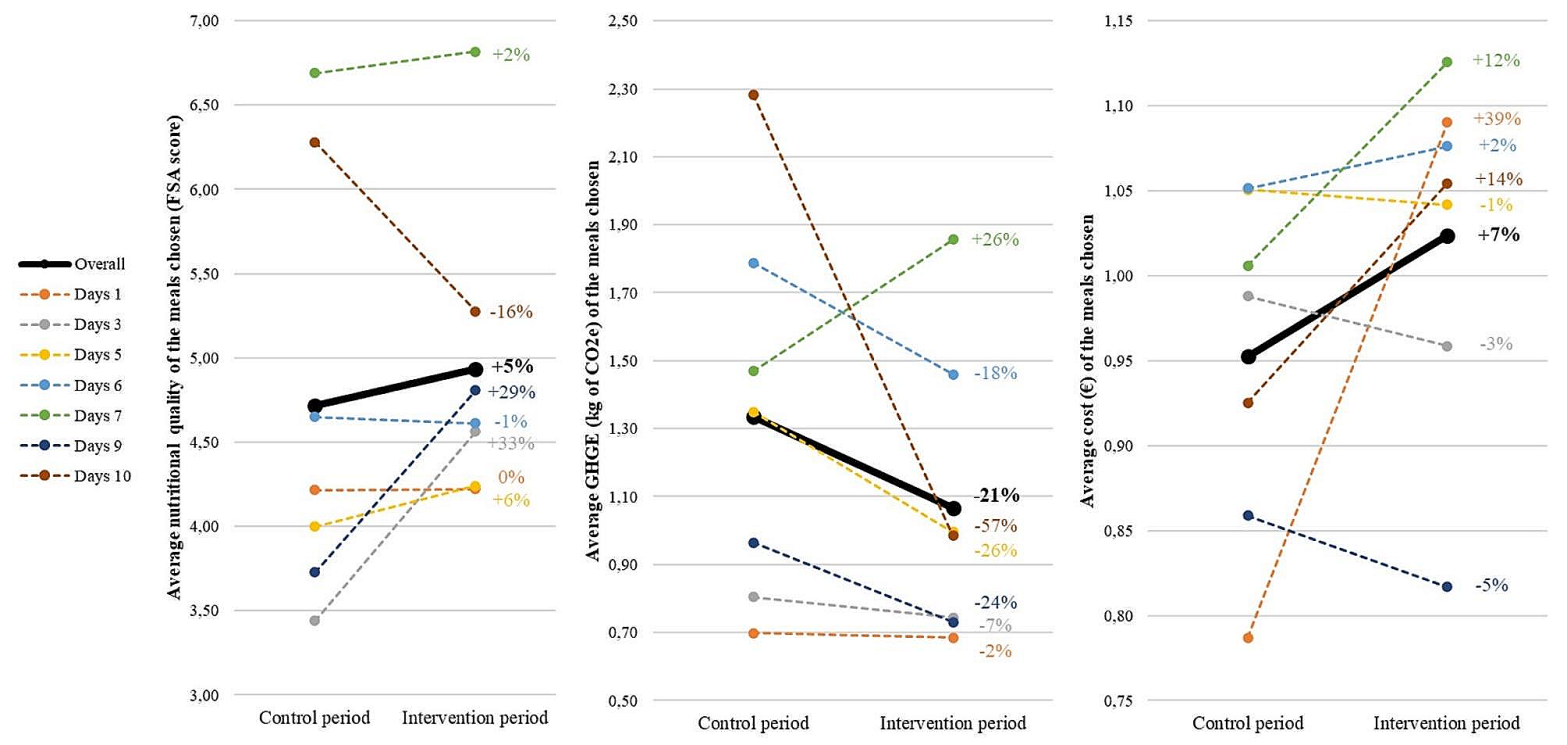
Sustainability indicators of food choices between control and intervention periods for the 7 lunchtime pairs

Doubling the availability of vegetarian main meals resulted in an increase in the FSA score of the main meals chosen (i.e., a decrease in nutritional quality) from 4.71 during the control period to 4.93 during the intervention period for an average main meal (β = 0.25, t(37,298) = 3.07, *p* = 0.002). GHGE decreased from 1.34 kg CO2 eq in the control period to 1.06 kg CO2 eq in the intervention period for an average main meal (β=-0.24, t(37,298)=-17.6, *p* < 0.001), i.e., 560 kg CO2 eq saved per day for 2000 guests. We also observed a slight cost increase from 0.95 € in the control period to 1.02€ in the intervention period for an average main meal (β = 0.08, t(37,298) = 20.3, *p* < 0.001), i.e., an increase of 140€ per day for 2,000 guests. Figure 5 depicts the variability in sustainability indicators across the seven pairs of lunchtimes studied.

### Perception of the intervention by the students

Among the 628 participants who started the feedback questionnaire, 506 were included in the analyses after the eligibility check (see flowchart, Fig. 2). Among the 506 students, 64% were female, 49% had scholarships, 31% were flexitarian, 7% were vegetarian or vegan and the average age was 20.6 ± 2.8 years. Additional sociodemographic details can be found in supplementary materials (S7). Only 5.5% (*n* = 28) of these students reported having noticed a change regarding vegetarian main meals during the intervention period. After being informed about this change in the food offered, 50% (*n* = 255) recalled having consumed more vegetarian main meals than usual and only 19% (*n* = 48) of them perceived this increase as a constraint (score > 5).

## Discussion

In this study, we assessed the effects of increasing vegetarian main meal availability on main meal choices while investigating how acceptable this intervention was in the real-life context of a university cafeteria. In a four-week experiment conducted in a French university cafeteria, pre-post analyses of students’ main meal choices demonstrated that doubling the availability of vegetarian main meals significantly increased the likelihood of vegetarian main meal choices. Moreover, compared to baseline, meal offer satisfaction and liking showed the high acceptability of vegetarian main meals availability interventions in this context. Furthermore, at the end of the experiment, only 5.5% of the students exposed to the intervention reported having noticed a change in the vegetarian main meals offered in this cafeteria.

From the analysis of 37,299 main meal choices, we observed that doubling the availability of vegetarian main meals from 24 to 48% led to a rise in vegetarian main meal choices from 23 to 45%; equivalent to an increase of 96%. The effect size is substantial and surpasses the results of similar studies conducted in British university cafeterias [24, 30]. A first study reported increases of 62%, 79% and 41% in three different cafeterias when the availability of vegetarian main meals was doubled from 25 to 50% [24]. A second natural experiment showed a 47% increase in vegetarian main meal choices when vegetarians’ availability doubled from 33 to 67% [30]. Differences in effect sizes between our study and those conducted in England may first be attributed to disparities in university food environments between England and France; for example, a lack of alternatives to the university cafeteria on the university campus in France might have made students more captive, leading to greater adherence to the offered food options. Second, the observed variations in effect size could be attributed to differences in the types of vegetarian main meal options offered in each intervention, particularly in terms of taste and familiarity. Indeed, in the present study, only existing cafeteria recipes (*n* = 63) were used; therefore, the students were familiar with the recipes.

This study aimed to examine participants’ meal offer satisfaction and main meal liking when availability of vegetarian meals was increaseds greater acceptability can enhance efficacy and increase the likelihood of adoption by institutions such as university cafeterias [39]. Contrary to our expectations, our results showed high acceptability of the intervention, with no decrease in satisfaction or liking. This outcome differs from the results of a survey of 2,215 British consumers, which suggested a declared consumer preference for information-based interventions over changes in food availability, as only 40% declared supporting interventions reducing meat availability [40]. Hence, consumers themselves may underestimate the acceptability of availability interventions. In our study, the sustained satisfaction may be attributed to not communicating the change in the food offered to the students. Indeed, people might be more likely to feel that their freedom of choice is limited when asked directly about their level of acceptability of such interventions, with freedom of choice being a fundamental element in the concept of accepting climate public policies [41]. When the availability of the food offered changes without drawing attention to the category of products targeted (i.e., vegetarian meals), participants might not necessarily notice or see it as a restriction as long as many options are offered. Also, in the present study, six meal options were offered throughout each service. Additionally, the results regarding the liking of chosen main meals in our study support that expanding options within a food category increases the likelihood of participants choosing a liked meal within this category [23, 28]. Finally, food waste data served as a corroborating measure for liking scores, showing no increase in food waste during the intervention period.

As secondary analyses, we examined the effect of the availability intervention targeting vegetarian main meals on sustainability indicators. Our findings revealed an overall reduction in the carbon footprint of the main meals chosen but a slight decrease in nutritional quality and an increase in cost of production. The results regarding GHGEs align with previous research indicating that vegetarian meals have a lower environmental impact than meat-based options [42, 43]. However, we did not expect to observe a decrease in nutritional quality, although previous studies highlighted that some vegetarian schools meals could be of low nutritional quality [44], or an increase in the cost of the main meals chosen during the intervention period. We observed some variability between the different pairs of lunchtimes studied, suggesting that individuals switching from a meat-based to a meat-free main meal did not always improve the nutritional quality, environmental impact, or cost of their meal choices. There is thus room to optimize menu planning to enhance meal choices sustainability when increasing vegetarian availability. In the present study, menus were created by the university cafeteria head chef with constraints on the vegetarian/nonvegetarian servings ratio but no guidelines on sustainability indicators. Therefore, foodservice professionals may be encouraged to calculate sustainability indicators based on their recipes to assist in designing menus that align with sustainability goals and to identify quick pathways toward a more sustainable meal offer.

### Strengths and limitations

This study has several strengths, including the preregistration of the protocol and analysis plan allowing for transparency and minimizing reporting bias. Conducted in a real-life setting, the experiment recorded a significant number of main meal choices (*n* = 37,299). Unique to this study, the measurement of interventions’ acceptability of satisfaction and liking was of high quality (large sample, good representation of the overall customer choices). Additionally, not communicating about the change in food offered may have limited bias regarding the demographics of the students visiting the university cafeteria during the intervention period, as we could argue that if students knew about the experiment, those who did not want to eat the vegetarian meal might have chosen to eat elsewhere. An additional strength was the design of the control period within the same university cafeteria, which maintained constant parameters of the food environment and lunch menus except for vegetarian meal availability.

However, there are limitations to consider. While the study aimed to control the food choice environment, the absence of a similar control cafeteria may have led to overlooking other contextual factors influencing vegetarian meal choices. However, given the limited duration of the intervention (2 weeks) and the size of the effect observed, we doubt that these differences could have been entirely attributed to factors other than availability. Another limitation is the number of strikes that happened during the data collection, which reduced the number of observed pairs of lunchtimes from 10 to 7; thus leading us not to perform one of our preregistered analyses. Furthermore, the data collection relied on production and sales data, and liking and satisfaction scores were anonymous, lacking identification of individual-level changes in food choices. We were not able to record what was available for whom at each time point during one lunchtime and our analyses rely on the average probability of vegetarian meals availability across one lunchtime. Finally, there may be limitations related to the length of the study, which lasted two weeks. Even if we observed a 96% increase in vegetarian meal selection, it remains uncertain whether this increase would have been sustainable over a longer duration or if there was a threshold of vegetarian main meal availability beyond which the choice no longer aligns with availability. To explore this phenomenon, additional observation days at various availability levels are necessary. Regarding the generalizability of the results, the study minimized selection bias by collecting food choice data from the entire student population eating in the study cafeteria. However, the limitation of data collection to a single university cafeteria in France raises concerns about external validity and replication in diverse settings would be essential. However, in the studied cafeteria we have good representation of the French student population in terms of sociodemographic characteristics.

## Conclusions

This study successfully replicated the effects of an availability intervention targeting vegetarian main meals in a French university cafeteria. We found a strong association between the availability of vegetarian main meals and vegetarian main meal choices revealing a pronounced influence of the food environment on university students’ food choices. As a novel contribution to the literature, we examined the acceptability of this intervention through a multidimensional assessment including students’ meal offer satisfaction and liking, which showed no negative effect of the increased availability of vegetarian meals. Additionally, we extended the evaluation of the impacts of the intervention beyond food choice outcomes to examine its effects on broader sustainability indicators. Doubling the availability of vegetarian main meals decreased the environmental impact of the main meal but led to a slight decrease in nutritional quality and a slight increase in cost of production. In summary, our results support the fact that availability interventions targeting vegetarian meals in university cafeterias offer a straightforward and highly acceptable way to enhance the choice of vegetarian main meals in the student population.

### Electronic supplementary material

Below is the link to the electronic supplementary material.

Supplementary Material 1

Supplementary Material 2

## Data Availability

The datasets analyzed during the current study will be made available on the Open Science Framework project page at the time of publication (https://osf.io/pf3x7/).

## Abbreviations

FAO: Food and Agriculture Organization of the United Nations
FSA score: British Food Standards Agency nutrient profiling system
GHGE: Greenhouse gas emissions
WHO: World Health Organization

## References

[1] Clark MA, Domingo NGG, Colgan K, Thakrar SK, Tilman D, Lynch J (2020). Global food system emissions could preclude achieving the 1.5° and 2°C climate change targets. Science.

[2] Willett W, Rockström J, Loken B, Springmann M, Lang T, Vermeulen S (2019). Food in the Anthropocene: the EAT–Lancet Commission on healthy diets from sustainable food systems. Lancet.

[3] Gakidou E, Afshin A, Abajobir AA, Abate KH, Abbafati C, Abbas KM (2017). Global, regional, and national comparative risk assessment of 84 behavioural, environmental and occupational, and metabolic risks or clusters of risks, 1990–2016: a systematic analysis for the global burden of Disease Study 2016. Lancet.

[4] Crippa M, Solazzo E, Guizzardi D, Monforti-Ferrario F, Tubiello FN, Leip A (2021). Food systems are responsible for a third of global anthropogenic GHG emissions. Nat Food.

[5] FAO and WHO. Sustainable healthy diets - Guiding principles. 2019; https://iris.who.int/bitstream/handle/10665/329409/9789241516648-eng.pdf?sequence=1

[6] FAO. Sustainable diets and biodiversity. Directions and solutions for policy, research and action. 2010. Report No.: 9789251072882.

[7] Rabès A, Seconda L, Langevin B, Allès B, Touvier M, Hercberg S (2020). Greenhouse gas emissions, energy demand and land use associated with omnivorous, pesco-vegetarian, vegetarian, and vegan diets accounting for farming practices. Sustainable Prod Consum.

[8] Perraud E, Wang J, Salomé M, Mariotti F (2023). Dietary protein consumption profiles show contrasting impacts on environmental and health indicators. Sci Total Environ.

[9] Fresán U, Sabaté J (2019). Vegetarian diets: Planetary Health and its alignment with Human Health. Adv Nutr.

[10] Graça J, Godinho CA, Truninger M (2019). Reducing meat consumption and following plant-based diets: current evidence and future directions to inform integrated transitions. Trends Food Sci Technol.

[11] Stoll-Kleemann S, Schmidt UJ (2017). Reducing meat consumption in developed and transition countries to counter climate change and biodiversity loss: a review of influence factors. Reg Envriron Chang.

[12] Marteau TM. Towards environmentally sustainable human behaviour: targeting non-conscious and conscious processes for effective and acceptable policies. Philosophical Trans Royal Soc A: Math Phys Eng Sci. 2017;375. 10.1098/rsta.2016.0371 10.1098/rsta.2016.0371 PMC5415649 28461435

[13] McGill R, Anwar E, Orton L, Bromley H, Lloyd-Williams F, O’Flaherty M (2015). Are interventions to promote healthy eating equally effective for all? Systematic review of socioeconomic inequalities in impact. BMC Public Health.

[14] Bianchi F, Dorsel C, Garnett E, Aveyard P, Jebb SA. Interventions targeting conscious determinants of human behaviour to reduce the demand for meat: a systematic review with qualitative comparative analysis. Int J Behav Nutr Phys Activity. 2018;15. 10.1186/s12966-018-0729-6 10.1186/s12966-018-0729-6 PMC6194670 30340498

[15] Bianchi F, Garnett E, Dorsel C, Aveyard P, Jebb SA (2018). Restructuring physical micro-environments to reduce the demand for meat: a systematic review and qualitative comparative analysis. Lancet Planet Health.

[16] Chang KB, Wooden A, Rosman L, Altema-Johnson D, Ramsing R (2023). Strategies for reducing meat consumption within college and university settings: a systematic review and meta-analysis. Front Sustain Food Syst.

[17] Carrington MJ, Neville BA, Whitwell GJ (2010). Why ethical consumers don’t walk their talk: towards a framework for understanding the gap between the ethical purchase intentions and actual buying behaviour of ethically minded consumers. J Bus Ethics.

[18] Carrington MJ, Neville BA, Whitwell GJ (2014). Lost in translation: exploring the ethical consumer intention-behavior gap. J Bus Res.

[19] Hollands GJ, Shemilt I, Marteau TM, Jebb SA, Kelly MP, Nakamura R, et al. Altering micro-environments to change population health behaviour: towards an evidence base for choice architecture interventions. BMC Public Health. 2013;13. 10.1186/1471-2458-13-1218 10.1186/1471-2458-13-1218 PMC3881502 24359583

[20] Hill JO, Peters JC (1998). Environmental contributions to the obesity epidemic. Science.

[21] Hollands GJ, Carter P, Anwer S, King SE, Jebb SA, Ogilvie D, et al. Altering the availability or proximity of food, alcohol, and tobacco products to change their selection and consumption. Cochrane Database Syst Reviews. 2019;9. 10.1002/14651858.CD012573.pub2 10.1002/14651858.CD012573.pub3 PMC6953356 31482606

[22] Marteau TM, Hollands GJ, Pechey R, Reynolds JP, Jebb SA. Changing the assortment of available food and drink for leaner, greener diets. BMJ. 2022;13(377). 10.1136/bmj-2021-069848 10.1136/bmj-2021-069848 35418445

[23] Pechey R, Hollands GJ, Carter P, Marteau TM (2020). Altering the availability of products within physical micro-environments: a conceptual framework. BMC Public Health.

[24] Garnett EE, Balmford A, Sandbrook C, Pilling MA, Marteau TM (2019). Impact of increasing vegetarian availability on meal selection and sales in cafeterias. Proc Natl Acad Sci USA.

[25] Ruby MB, Alvarenga MS, Rozin P, Kirby TA, Richer E, Rutsztein G (2016). Attitudes toward beef and vegetarians in Argentina, Brazil, France, and the USA. Appetite.

[26] Melendrez-Ruiz J, Chambaron S, Buatois Q, Monnery-Patris S, Arvisenet G (2019). A central place for meat, but what about pulses? Studying French consumers’ representations of main dish structure, using an indirect approach. Food Res Int.

[27] Magrini M-B, Fernandez-Inigo H, Doré A, Pauly O (2021). How institutional food services can contribute to sustainable agrifood systems? Investigating legume-serving, legume-cooking and legume-sourcing through France in 2019. Rev Agric Food Environ Stud.

[28] Pechey R, Hollands GJ, Marteau TM (2022). Explaining the effect on food selection of altering availability: two experimental studies on the role of relative preferences. BMC Public Health.

[29] White M, Kwasnicka D, Dombrowski SU, White M (2016). Theoretical explanations for maintenance of behavior change: a systematic review of behavior theories. Health Psychol Rev.

[30] Pechey R, Bateman P, Cook B, Jebb SA (2022). Impact of increasing the relative availability of meat-free options on food selection: two natural field experiments and an online randomised trial. Int J Behav Nutr Phys Activity.

[31] Stiles G, Collins J, Beck KL (2022). Effectiveness of strategies to decrease animal-sourced protein and/or increase plant-sourced protein in foodservice settings: a systematic literature review. J Acad Nutr Dietetics.

[32] Sekhon M, Cartwright M, Francis JJ (2017). Acceptability of healthcare interventions: an overview of reviews and development of a theoretical framework. BMC Health Serv Res.

[33] Derqui B, Fernandez V (2017). The opportunity of tracking food waste in school canteens: guidelines for self-assessment. Waste Manag.

[34] József Tóth A, Dunay A, Bálint Illés C, Battay M, Bittsánszky A, Süth M (2023). Food liking and consumption in schools: comparison of questionnaire-based surveys with real consumption. Food Qual Prefer.

[35] Rayner M, Scarborough P, Lobstein T, The, UK Ofcom Nutrient Profiling Model. Defining Healthy and Unhealthy Foods and Drinks for TV Advertising to Children. 2009 https://www.ndph.ox.ac.uk/cpnp/files/about/uk-ofcom-nutrient-profile-model.pdf. Accessed 20 April 2023.

[36] The Scientific Committee of the Nutri-Score. Update report from the Scientific Committee of the Nutri-Score. 2022. https://www.aesan.gob.es/AECOSAN/docs/documentos/Nutri_Score/2022_main_algorithm_report_update_FINAL.pdf. Accessed 20 April 2023.

[37] Zampori L, Pant R. Suggestions for updating the Product Environmental Footprint (PEF) method. Publ. Off. Eur. Union. Luxembourg; 2019. https://ec.europa.eu/jrc

[38] ADEME. Agribalyse v3.0. 2020. https://ecolab.ademe.fr/agribalyse

[39] Diepeveen S, Ling T, Suhrcke M, Roland M, Marteau TM (2013). Public acceptability of government intervention to change health-related behaviours: a systematic review and narrative synthesis. BMC Public Health.

[40] Pechey R, Reynolds JP, Cook B, Marteau TM, Jebb SA (2022). Acceptability of policies to reduce consumption of red and processed meat: a population-based survey experiment. J Environ Psychol.

[41] Bendz A, Bäckstedt F, Harring N, Martin Persson U (2023). Why do people accept or reject climate policies targeting food consumption? Unpacking justifications in the public debate in online social forums. Food Policy.

[42] Dahmani J, Nicklaus S, Marty L. Nutritional quality and greenhouse gas emissions of vegetarian and non-vegetarian school meals: a case study in France (Dijon). Front Nutr. 2022;81. 10.3389/fnut.2022.997144 10.3389/fnut.2022.997144 PMC9590375 36299986

[43] Poinsot R, Vieux F, Maillot M, Darmon N (2022). Number of meal components, nutritional guidelines, vegetarian meals, avoiding ruminant meat: what is the best trade-off for improving school meal sustainability?. Eur J Nutr.

[44] Poinsot R, Vieux F, Dubois C, Perignon M, Méjean C, Darmon N. On behalf of the EnScol Network. Nutritional quality of vegetarian and non-vegetarian dishes at School: are nutrient profiling systems sufficiently informative? Nutrients. 2020; *12*(8), 2256. 10.3390/nu12082256 10.3390/nu12082256 PMC7468702 32731494

