# Supplementary material for: Effects of increasing the availability of vegetarian options on main meal choices, meal offer satisfaction and liking: a pre-post analysis in a French university cafeteria
Source: Int J Behav Nutr Phys Act. 2024 Jul 16;21:75. doi: 10.1186/s12966-024-01624-4 (PMC11250975; doi:10.1186/s12966-024-01624-4)
Supplement: Supplementary file 1 — Supplementary Material 1 [file 12966_2024_1624_MOESM1_ESM.docx]

**Supplementary materials**

| **Effects of increasing the availability of vegetarian options on meal choices, meal offer satisfaction and liking: A pre-post analysis in a French university cafeteria** |
| --- |

**Laura Arrazat^a^, Claire Cambriels^a^, Christine Le Noan^b^, Sophie Nicklaus^a^, Lucile Marty^a^**

^a^ Centre des Sciences du Goût et de l'Alimentation, CNRS, INRAE, Institut Agro, Université de Bourgogne, France

^b^ Crous Bourgogne Franche-Comté, France

**S1.** **Main meals served during the control and intervention periods**

| **Lunchtime** | **Control period** | | **Intervention period** | |
| --- | --- | --- | --- | --- |
|  | ***Name of the main meal*** | ***Number of servings*** | ***Name of the main meal*** | ***Number of servings*** |
| 1 | Cordon bleu | 400 | Cordon bleu | 320 |
|  | Pork with pepper sauce | 500 | Ham with mushrooms and cream | 360 |
|  | **Omelette** | 600 | **Cheese omelette** | 540 |
|  | Carbonara pasta | 320 | **Cheese tortellini** | 600 |
|  | Ham pizza | 600 | Ham pizza | 540 |
|  | Spring rolls | 300 | **Vegetable Samosa** | 280 |
| 2 |  | | Ground beef steak | 288 |
|  |  |  | Fish stew in white sauce | 480 |
|  |  |  | **Vegetarian burger** | 456 |
|  |  |  | **Vegetarian wheat and spinach steak** | 500 |
|  |  |  | Meat couscous | 422 |
|  |  |  | Chorizo pizza | 540 |
|  |  |  | Nuggets | 100 |
| 3 | Creole-style pork sauté | 440 | Creole-style pork sauté | 350 |
|  | Poultry sausage | 300 | Poultry sausage | 240 |
|  | **Tomato and basil vegetarian steak** | 600 | **Tomato and basil vegetarian steak** | 360 |
|  | Kebab pizza | 600 | **Greek pizza** | 500 |
|  | Pasta with Spanish sauce (chorizo) | 220 | Pasta with Spanish sauce (chorizo) | 300 |
|  | Jambalaya chicken | 320 | Jambalaya chicken | 400 |
|  |  |  | **Thai wheat balls** | 66 |
|  |  |  | Nuggets | 120 |
| 4 | Tex-Mex chicken drumsticks | 728 |  | |
|  | **Chickpea curry** | 480 |  |  |
|  | Ground beef steak | 288 |  |  |
|  | Bolognese pizza | 475 |  |  |
|  | Fish with tomato sauce | 380 |  |  |
|  | Chili con carne | 400 |  |  |
| 5 | Stuffed veal roast | 350 | Stuffed veal roast | 325 |
|  | Grilled pork escalope | 225 | **Vegetarian lasagna** | 720 |
|  | Bolognese lasagna | 432 |  |  |
|  | **Margherita pizza** | 400 | **Margherita pizza** | 400 |
|  | **Thai wheat balls** | 286 | **Wheat tenders** | 240 |
|  | Calamari | 360 | Calamari | 225 |
|  | Spring rolls | 48 |  |  |
|  | Nuggets | 20 |  |  |
| 6 | Ground beef steak | 336 | Ground beef steak | 384 |
|  | **Omelette with mixed vegetables** | 475 | **Omelette with mixed vegetables** | 400 |
|  | Pizza with chorizo | 570 | **Margherita pizza** | 450 |
|  | Hachis parmentier | 363 | Hachis parmentier | 363 |
|  | Pasta with Amatriciana sauce | 320 | Pasta with Amatriciana sauce | 300 |
|  |  |  | Stuffed zucchinis | 512 |
|  |  |  | Pizza with chorizo | 150 |
| 7 | **Vegetarian chili** | 550 | **Vegetarian chili** | 200 |
|  | Chicken stew in white sauce | 650 | Chicken stew in white sauce | 575 |
|  | Ground beef steak | 360 | **Tomato and basil vegetarian steak** | 390 |
|  | Flammekueche pizza | 570 | Flammekueche pizza | 600 |
|  | Cheese burger | 432 | Cheese burger | 408 |
|  | Chicken Garam Masala | 250 | **Vegetarian moussaka** | 280 |
| 8 |  | | Ground beef steak | 312 |
|  |  |  | Flammekueche pizza | 110 |
|  |  |  | Caramelized pork | 250 |
|  |  |  | **Cheese puff pastry** | 350 |
|  |  |  | **Mushroom gnocchi** | 390 |
|  |  |  | **Blue cheese and goat cheese pizza** | 380 |
|  |  |  | Roasted chicken drumsticks | 405 |
|  |  |  | Nuggets | 40 |
|  |  |  | **Thai wheat balls** | 40 |
| 9 | Cordon bleu | 320 | Cordon bleu | 300 |
|  | **Thai wheat balls** | 506 | **Thai wheat balls** | 429 |
|  | Tuna pizza | 465 | Tuna pizza | 450 |
|  | Obernai-style cervelas | 380 | Obernai-style cervelas | 360 |
|  | Pork shoulder | 400 | **Vegetarian wheat and spinach steak** | 375 |
|  | Turkey slices with curry sauce | 300 | Turkey slices with curry sauce | 350 |
|  |  |  | Nuggets | 80 |
| 10 | Ground beef steak | 346 | Ground beef steak | 336 |
|  | Lamb meatballs with cumin | 330 | **Provencal tart** | 530 |
|  | Breaded fish | 340 | Breaded fish | 280 |
|  | Caramelized pork | 300 |  |  |
|  | **Lentil Dahl** | 240 | **Lentil Dahl** | 475 |
|  | **Goat cheese and vegetable pizza** | 480 | **Goat cheese and vegetable pizza** | 430 |

*Green text signals vegetarian options. Grey zones: unavailable data (strikes)*

**S2. Co-construction procedure**

**STEP 1. Analysis of the sustainability of the main meals served in the university cafeteria over a full school year (September 2021 - May 2022)**

We conducted a quantitative analysis of the nutritional quality (FSA score), environmental impact (GHGE), and cost (€) of the main meals served throughout an entire school year (n=178 meals). These main meals were categorized into four groups: red meat, poultry or pork, fish, and vegetarian meals. Moreover, we simulated the potential advantages of increasing the availability of vegetarian meals across these three sustainability indicators (results not shown).

We completed this quantitative analysis with 7 semi-structured interviews with members of the staff of the university cafeteria. These interviews had three objectives. First to gain a better understanding of the functioning of the university cafeteria, particularly in terms of decision-making responsibilities. The second goal was to understand how members of the university cafeteria perceived the concept of sustainability. Lastly, we aimed to identify barriers and drivers for enhancing the sustainability of the food served in the university cafeteria.

**STEP 2. Collective choice of a structural intervention**

The findings from this diagnosis were presented to the university cafeteria management. Based on the observation of the low availability of vegetarian meals, we proposed to run a structural intervention of the availability of vegetarian meals. One of the concerns expressed by the management was the potential negative response from students. Therefore, we decided to initiate the present experiment to assess the acceptability of an intervention aimed at modifying the availability of vegetarian options among the student population.

**STEP 3. Setting up of a working group**

To design and carry out this experiment, we established a working group comprised of five key members from the university cafeteria staff. The following members were included: the Dining Services Manager, responsible for personnel management; the Sustainability Manager, who specializes in measuring food waste; the dietitian, contributing to menu planning; the Head Chef of the university cafeteria, overseeing menu decisions and kitchen organization; and the Assistant Chef, responsible for task allocation in the kitchen.

The choice of these five members were based on results from the qualitative interviews conducted with them in the year preceding the present study (STEP 1) which allowed us to grasp a better understanding of their roles.

**STEP 4. Working group sessions**

- **Session 1. Design and objectives of the experiment.**

The objective of this initial session was to collaboratively determine the experimental design that would be implemented. As project scientists, we presented the ideal experimental design to minimize biases, particularly what we would have done in the context of a laboratory experiment. Subsequently, we engaged in discussions regarding what was feasible or not based on field constraints. Consequently, we made decisions regarding the duration of the experiment, the type of design (controlled trial), and the parameters that could be controlled in the context of this university cafeteria.

- **Session 2. Evaluation of the intervention.**

During the second session, we discussed the evaluation procedures for the intervention's effects. In this regard, we collectively identified the variables of interest and discussed how each variable could be effectively measured. Finally, we allocated roles for data collection among the team members.

**S3. Satisfaction and liking paper ballots**

**
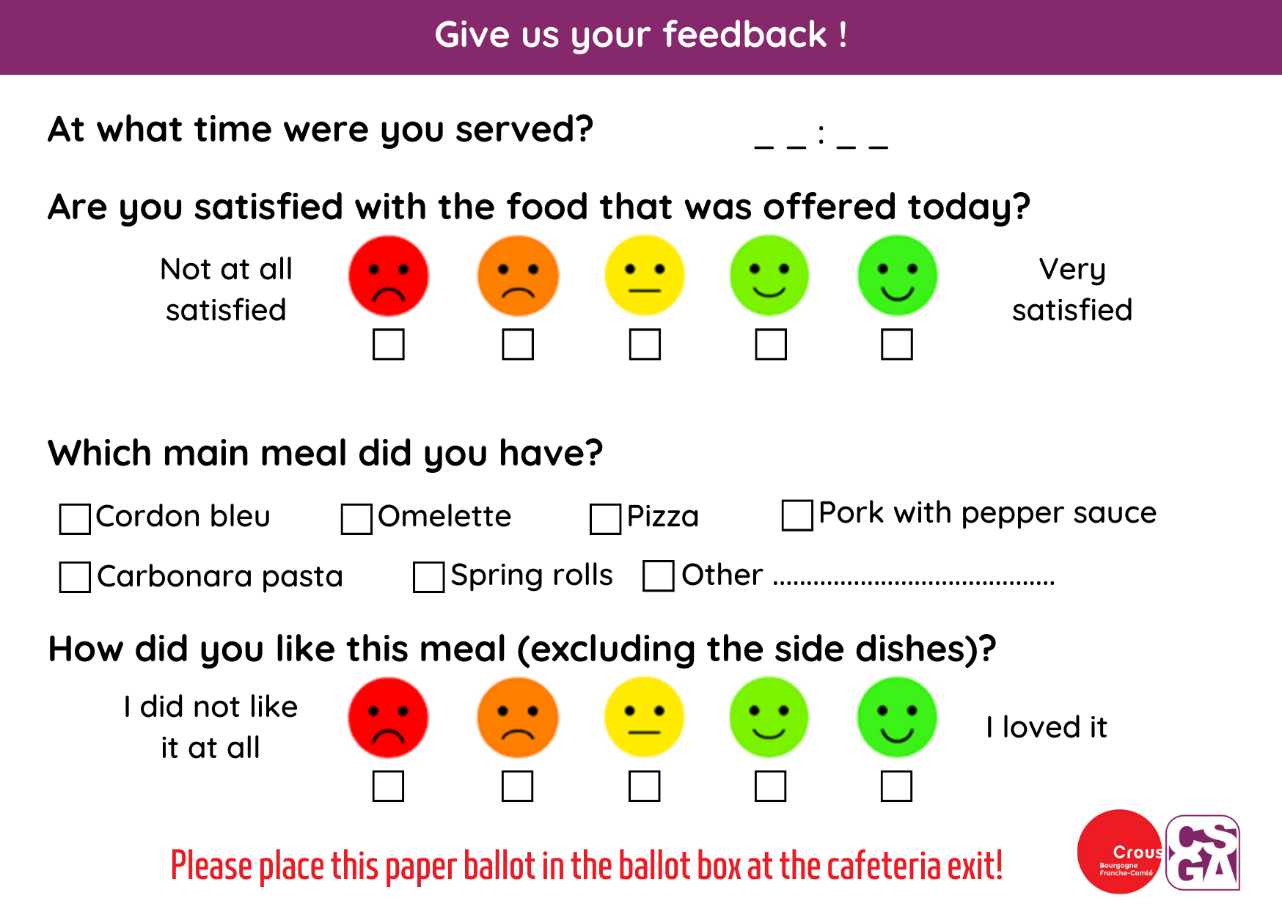
**

**S4. Sustainability indicators for the 63 main meals**

| **Main meals served during the study** | **Nutritional quality** | **Environmental impact** | **Cost** | **Liking score** | |
| --- | --- | --- | --- | --- | --- |
|  | ***FSA score/100g*** | ***GHGE in kg CO2 eq/serving*** | ***€/serving*** | ***Mean [1; 5]*** | ***SD*** |
| Bolognese lasagna | 2,00 | 2,87 | 1,82 | 4,68 | 0,56 |
| Caramelized pork | -1,00 | 0,95 | 0,91 | 4,56 | 0,62 |
| **Vegetarian lasagna** | 0,50 | 0,97 | 1,27 | 4,49 | 0,76 |
| **Greek pizza** | 4,50 | 0,72 | 1,18 | 4,42 | 0,82 |
| Jambalaya chicken | -2,00 | 1,05 | 1,25 | 4,42 | 0,79 |
| Turkey slices with curry sauce | -3,00 | 0,59 | 0,85 | 4,39 | 0,75 |
| Cheese burger | 17,00 | 5,49 | 1,69 | 4,36 | 0,76 |
| Ham with mushrooms and cream | 0,00 | 1,01 | 1,20 | 4,34 | 0,74 |
| **Chickpea curry** | 0,00 | 0,47 | 1,03 | 4,31 | 0,85 |
| Roasted chicken drumsticks | -1,00 | 1,14 | 0,82 | 4,30 | 0,81 |
| Chorizo pizza | 15,00 | 1,05 | 1,19 | 4,29 | 1,24 |
| Hachis parmentier | 2,00 | 2,76 | 1,65 | 4,29 | 0,74 |
| **Lentil Dahl** | -8,00 | 0,24 | 0,63 | 4,27 | 0,96 |
| **Blue cheese and goat cheese pizza** | 17,00 | 0,93 | 1,43 | 4,26 | 0,89 |
| Turkey with curry sauce | -6,50 | 0,74 | 0,98 | 4,26 | 0,79 |
| Chicken stew in white sauce | -5,00 | 0,71 | 0,84 | 4,25 | 0,77 |
| Creole-style pork sauté | 0,00 | 1,12 | 0,97 | 4,25 | 0,76 |
| Stuffed zucchinis | -0,50 | 0,70 | 0,91 | 4,25 | 0,71 |
| Ham pizza | 4,00 | 0,76 | 0,94 | 4,24 | 0,78 |
| Lamb meatballs with cumin | 11,00 | 7,58 | 0,74 | 4,23 | 0,94 |
| Pizza with chorizo | 12,50 | 3,17 | 1,06 | 4,22 | 0,70 |
| **Goat cheese and vegetable pizza** | 11,50 | 0,63 | 1,00 | 4,22 | 0,89 |
| Bolognese pizza | 4,50 | 1,32 | 1,10 | 4,21 | 0,90 |
| Chili con carne | -0,50 | 3,03 | 1,69 | 4,21 | 0,75 |
| **Margherita pizza** | 9,50 | 0,59 | 0,98 | 4,21 | 0,88 |
| Calamari | 12,00 | 0,82 | 0,51 | 4,21 | 0,84 |
| Kebab pizza | 1,00 | 0,98 | 1,18 | 4,17 | 0,83 |
| Ground beef steak | 7,00 | 2,43 | 1,22 | 4,17 | 0,83 |
| Flammekueche pizza | 17,00 | 1,02 | 1,18 | 4,16 | 0,87 |
| Cordon bleu | 13,00 | 0,64 | 0,52 | 4,16 | 0,78 |
| Tex-Mex chicken drumsticks | -3,00 | 1,02 | 0,99 | 4,15 | 0,85 |
| Meat couscous | 4,00 | 5,25 | 1,89 | 4,15 | 0,86 |
| Pork with pepper sauce | -1,00 | 1,22 | 0,86 | 4,14 | 0,71 |
| Tuna pizza | 1,00 | 1,03 | 1,09 | 4,13 | 0,96 |
| **Vegetarian burger** | 11,00 | 1,02 | 1,06 | 4,10 | 0,97 |
| Fish stew in white sauce | 3,00 | 2,01 | 1,22 | 4,09 | 0,92 |
| Pasta with Amatriciana sauce | 4,50 | 0,71 | 0,84 | 4,08 | 0,83 |
| **Cheese omelette** | 1,00 | 0,47 | 0,81 | 4,08 | 0,85 |
| **Vegetarian moussaka** | -5,00 | 1,30 | 1,06 | 4,07 | 1,07 |
| Breaded fish | 4,00 | 1,04 | 0,81 | 4,05 | 0,88 |
| **Provencal tart** | 3,50 | 0,53 | 1,25 | 4,05 | 0,97 |
| Carbonara pasta | 14,00 | 0,94 | 1,01 | 4,04 | 0,75 |
| **Cheese tortellini** | 2,50 | 0,86 | 2,20 | 4,02 | 0,77 |
| Stuffed veal roast | -1,50 | 2,17 | 1,16 | 4,00 | 0,96 |
| Pasta with Spanish sauce (chorizo) | 12,50 | 0,49 | 0,86 | 3,99 | 0,91 |
| **Vegetarian chili** | -2,50 | 0,24 | 0,45 | 3,99 | 1,05 |
| **Vegetarian wheat and spinach steak** | 5,00 | 0,14 | 0,63 | 3,98 | 1,05 |
| Pork shoulder | -4,00 | 1,85 | 1,04 | 3,96 | 0,97 |
| **Tomato and basil vegetarian steak** | -0,50 | 0,07 | 1,00 | 3,96 | 0,98 |
| While fish with tomato sauce | 2,00 | 1,22 | 0,90 | 3,93 | 0,99 |
| **Wheat tenders** | -3,50 | 0,17 | 0,90 | 3,84 | 1,09 |
| **Vegetable Samosa** | 11,00 | 0,29 | 0,61 | 3,83 | 1,07 |
| **Cheese puff pastry** | 21,00 | 0,60 | 0,97 | 3,82 | 1,10 |
| Obernai-style cervelas | 21,00 | 1,58 | 0,77 | 3,81 | 1,02 |
| **Omelette with mixed vegetables** | 0,00 | 0,39 | 0,69 | 3,79 | 1,03 |
| **Wheat tenders** | 5,00 | 0,17 | 0,85 | 3,7 | 1,05 |
| **Omelette with pepper** | -1,00 | 0,42 | 0,70 | 3,70 | 1,09 |
| Fricadelle | 22,00 | 0,91 | 1,10 | 3,69 | 1,07 |
| Poultry sausage | 18,00 | 1,36 | 0,47 | 3,65 | 1,05 |
| **Mushroom gnocchi** | 4,50 | 0,51 | 1,31 | 3,59 | 1,12 |
| Grilled pork escalope | -3,00 | 1,22 | 0,99 | 3,58 | 0,99 |
| Spring rolls | 4,00 | 0,26 | 0,71 | 3,58 | 0,87 |
| **Thai wheat balls** | 1,00 | 0,11 | 0,70 | 3,50 | 1,22 |

*Green text signals vegetarian main meals.*

**S5. Descriptive statistics of daily satisfaction scores**

| **Meal day** | **Intervention** | **N** | **Average** | **Standard deviation** |
| --- | --- | --- | --- | --- |
| 1 | 0 | 1175 | 4.0 | 0.9 |
| 3 | 0 | 1183 | 4.0 | 0.8 |
| 4 | 0 | 1205 | 4.1 | 0.9 |
| 5 | 0 | 960 | 3.9 | 1.1 |
| 6 | 0 | 1212 | 4.1 | 0.8 |
| 7 | 0 | 1298 | 4.1 | 0.9 |
| 9 | 0 | 1118 | 4.0 | 1.0 |
| 10 | 0 | 850 | 4.1 | 1.0 |
| 1 | 1 | 1216 | 4.1 | 0.9 |
| 2 | 1 | 1366 | 4.1 | 1.0 |
| 3 | 1 | 1161 | 4.1 | 0.9 |
| 5 | 1 | 830 | 4.2 | 0.9 |
| 6 | 1 | 1039 | 4.0 | 0.9 |
| 7 | 1 | 780 | 4.2 | 0.9 |
| 8 | 1 | 1054 | 4.1 | 0.9 |
| 9 | 1 | 1066 | 3.9 | 1.0 |
| 10 | 1 | 802 | 4.1 | 1.0 |

**S6. Descriptive statistics of daily liking scores**

| **Meal day** | **Intervention** | **N** | **Average** | **Standard deviation** |
| --- | --- | --- | --- | --- |
| 1 | 0 | 1172 | 3.9 | 0.9 |
| 3 | 0 | 1173 | 4.1 | 0.9 |
| 4 | 0 | 1189 | 4.2 | 0.9 |
| 5 | 0 | 942 | 4.0 | 1.1 |
| 6 | 0 | 1190 | 4.1 | 0.8 |
| 7 | 0 | 1272 | 4.2 | 0.9 |
| 9 | 0 | 1096 | 4.0 | 0.9 |
| 10 | 0 | 837 | 4.3 | 0.9 |
| 1 | 1 | 1191 | 4.1 | 0.9 |
| 2 | 1 | 1352 | 4.1 | 0.9 |
| 3 | 1 | 1146 | 4.2 | 0.9 |
| 5 | 1 | 823 | 4.3 | 0.9 |
| 6 | 1 | 1015 | 4.1 | 0.9 |
| 7 | 1 | 758 | 4.2 | 0.9 |
| 8 | 1 | 1038 | 4.0 | 1.0 |
| 9 | 1 | 1045 | 4.0 | 1.0 |
| 10 | 1 | 793 | 4.2 | 0.9 |

**S7. Sociodemographic characteristics of participants of the feedback questionnaire (N=506)**

| **Age**, *years*, mean (SD) | 20,6 (2,8) |
| --- | --- |
| **Sex**, n (%) |  |
| Female | 322 (64%) |
| Male | 178 (35%) |
| Others | 6 (1%) |
| **Scholarship status**, *with scholarship*, n (%) | 248 (49%) |
| **Type of institution** ^a^**,** n (%) |  |
| University | 405 (80%) |
| Others | 101 (20%) |
| **Field of studies** ^b^, n (%) |  |
| Science | 122 (24%) |
| Humanities | 384 (76%) |
| **Highest educational qualification**, n (%) |  |
| < High-school +1 years diploma | 132 (26%) |
| High-school +2 years diploma | 136 (27%) |
| High-school +3 years diploma | 119 (24%) |
| High-school +4 years diploma | 66 (13%) |
| High-school +5 years diploma | 30 (6%) |
| ≥ High-school +6 years diploma | 23 (5%) |
| **Place of living**, n (%) |  |
| Parents’ house | 103 (20%) |
| Students’ residence | 88 (17%) |
| Independent accommodation | 315 (62%) |
| **Declared diet**, n (%) |  |
| Omnivore | 314 (62%) |
| Flexitarian | 157 (31%) |
| Pesco-vegetarian | 15 (3%) |
| Ovo-lacto-vegetarian | 18 (4%) |
| Vegan | 2 (1%) |
